# Coalescence of multiple pairs of levitated droplets using dual-side phased arrays

**DOI:** 10.1016/j.ultsonch.2025.107327

**Published:** 2025-03-31

**Authors:** Jianqing Li, Nicholas J. Goddard, Ruamsiri Songsaeng, Ruchi Gupta

**Affiliations:** School of Chemistry, University of Birmingham, Birmingham B15 2TT, UK

**Keywords:** Acoustic, Levitation, Coalescence, Droplets, Containerless, Reactions

## Abstract

Acoustic levitation in air and contactless coalescence of levitated droplets using acoustic forces are of great significance to chemical and biological reactions. The state-of-the-art is levitation and coalescence of 3 pairs of droplets achieved via dual-side phased arrays. However, there are no reports on the general design principles for manipulation and coalescence of > 3 pairs of droplets. Equally, there are no reports on sequential coalescence of more than two columns of droplets, which is essential for performing reactions requiring addition of more than two reagents. In this paper, we showed that wide traps are more suited than narrow traps for the coalescence of droplets. In wide traps, the acoustic energy was expanded along the direction of merging of droplets. Additionally, uniform traps created in this work by distributing energy between traps increased the number of droplets that can be levitated. We have reported a new algorithm named DS-PAT based on direct search method to overcome the limitations of existing algorithms. Using wide uniform traps and the DS-PAT algorithm, for the first time, a stable coalescence of up to 6 pairs of levitated droplets was achieved. To measure experimental acoustic fields during the merging process, a custom-built acoustic scanning setup was employed, which showed good consistency with simulations. Subsequently, DS-PAT was used to design the sequential coalescence of 4 columns of droplets with 2 droplets in each column. This was then applied to study the well-known oscillatory Belousov–Zhabotinsky (BZ) reaction. This work gives general principles of designing acoustic fields for stable coalescence of columns of droplets and introduces a global algorithm for dual-side phased arrays, paving the way for stable and efficient chemical and biological reactions in airborne droplets.

## 1 Introduction

Acoustic levitation has numerous applications in the manipulation of particles [1], including droplets for containerless chemical reactions [2], liquid-marble micro-reactions [3], rheometry [4], X-ray diffraction samples [5], and the amorphization of pharmaceuticals [6]. The ability to manipulate droplets acoustically, especially translation followed by coalescence of multiple droplets is key for containerless chemical reactions. Typically, levitators capable of generating acoustic forces sufficient to levitate objects with density comparable to water, can be constructed using either a single large emitter [[7], [8], [9]] or phased array transducers [10]. An example of a large emitter is the Langevin horn, which has been used to levitate some of the densest liquids (such as mercury) and solids (such as iridium) on Earth [11], as well as an expanded polystyrene sphere of diameter [12] that is nearly three times the ultrasound wavelength. Several horns arranged along a line and operating in synergy have also been used to merge droplets [8]. In this case, typically the 2 nearest horns contributed to the acoustic force for the translation of droplets. The relative voltage of the 2 nearest horns was adjusted to translate and coalesce a pair of droplets. While Langevin horns are highly efficient on electroacoustic conversion, they are sensitive to changes in temperature, relative arrangement of elements forming the horns, and also need high input voltage typically, 100–1000 V [13]. These limitations can be overcome by phased arrays of low-cost transducers while allowing highly flexible and precise programmable control of focal points for trapping and manoeuvering of droplets [13]. For example, Songsaeng *et al.* used phased arrays to levitate up to 5 droplets in a column to investigate the Belousov-Zhabotinsky (BZ) oscillating reaction [2]. Hasegawa *et al.* used phased arrays to study the edge deformation of two interacting droplets during coalescence [14]. They also showed that droplets larger than the half-wavelength can be levitated under reduced gravity [15]. The active deformation of levitated droplets in oscillation mode has been used to promote mixing [16], which can be challenging in closed-channel droplet microfluidics. Vashi *et al.* studied the vertical coalescence of droplets with different surface tension, identifying three possible outcomes: partial merging, complete merging and core–shell bead formation, and found that parameters such as the characteristic size after coalescence and impact velocity of the upper droplet can influence the outcome of coalescence [17]. Gupta *et al.* have already realized the coalescence of up to three pairs of levitated droplets by manipulating two columns each trapping three droplets and conducted parallel enzyme assays [18], which is a notable improvement compared to the previous limit of two droplets [8,[14], [15], [16], [17]].

While the active coalescence of up to 3-pairs of droplets using phased arrays has been achieved [18], there is still a significant challenge of merging multiple pairs of droplets by two-side phased arrays. The challenges include generating multiple traps with uniform acoustic power that can levitate droplets and then coalescing them without loss or atomization [18,19]. Simultaneous merging of multiple pairs of droplets requires that they can be stably levitated, translated and coalesced. For the stable levitation of multiple pairs of droplets, the acoustic energy should be uniformly distributed into multiple pairs of focal points at first. For stable translation and coalescence, pressure variation should be carefully designed. Equally, there are no reports on sequential coalescence of more than two columns of droplets, which is essential for performing reactions requiring sequential addition of more than two reagents. In this case, the challenges include creating multiple pairs of focal points that can be manipulated independently. Another challenge is to ensure that while droplets in selected pairs of focal points are translated and coalesced, droplets in other focal points do not fall.

Up to now, many algorithms have been developed to optimize the phases of transducer arrays. The algorithms developed so far include the simple Checkerboard method which divides the alternating transducers into odd and even elements [20], iterative back propagation (IBP) algorithm [21], and Diff-PAT algorithm [22,23]. Limitations of current algorithms include limited accuracy and flexibility as well as inability to find optimum solutions for manipulation of droplets. For example, the Checkerboard method is less accurate because it assumes that each focal point is determined by odd or even transducers separately and ignores the interaction between the odd and even transducers. Furthermore, it is limited to the design of 2 foci, which in turn means that reactions requiring sequential addition of more than two reagents cannot be performed. IBP belongs to a series of phase retrieval algorithms originating from the Gerchberg-Saxton algorithm (GS) used for determining the amplitude and phase of optical wave functions [24] and is easily trapped in the local minima. Diff-PAT is a gradient-based algorithm [25] and can achieve the reconstruction of specific pressure levels compared to IBP and Checkerboard to a certain extent, but is still not a global algorithm.

Not only is the algorithm itself important, but the design principles are crucial for the stable coalescence of droplets both in parallel and sequentially. The optimization algorithms above are not intended for the coalescence of droplets, and hence approaches for using these algorithms to achieve coalescence of multiple droplets have not been reported. In this paper, stable coalescence of up to six pairs of levitated droplets was achieved by our newly developed algorithm DS-PAT based on direct search method in acoustic hologram design [26,27]. DS-PAT could successfully find an acoustic field with up to 6 uniform nodes followed by wide traps for simultaneous coalescence of 6 pairs of droplets, which has never been achieved by the previous algorithms. We showed that wide traps are more stable than narrow traps in the coalescence of droplets. This means that the acoustic energy should be expanded along the merging direction to an extent to realize stable coalescence. To record the experimental acoustic fields during the merging process, a custom-built acoustic scanning setup was employed to obtain the shape of whole acoustic field. We showed good consistency between experimental acoustic fields and those obtained via simulations. Subsequently, we used DS-PAT to design the sequential coalescence of 4 pairs of droplets which was then applied for sequential merging of 4 reagents to start the well-known oscillatory Belousov–Zhabotinsky (BZ) reaction in a completely automated and non-contact fashion [2,[28], [29], [30]].

## 2 Experimental

### 2.1 Chemicals

Amaranth (A1016-50G), Sulfuric acid (H_2_SO4), malonic acid (CH_2_(COOH)_2_), potassium bromate (KBrO_3_), potassium bromide (KBr), cerium (IV) ammonium nitrate (Ce(NH_4_)_2_(NO_3_)_6_), iron(II) sulphate heptahydrate (FeSO_4_·7H_2_O), and 1,10-phenanthroline were purchased from Sigma Aldrich Ltd (Gillingham, Dorset, UK). Phosphate-buffered saline (PBS, 10X), pH 7.4 was purchased from Thermo Fisher (Loughborough, UK).

### 2.2 Instrumentation

**Fig. 1:**
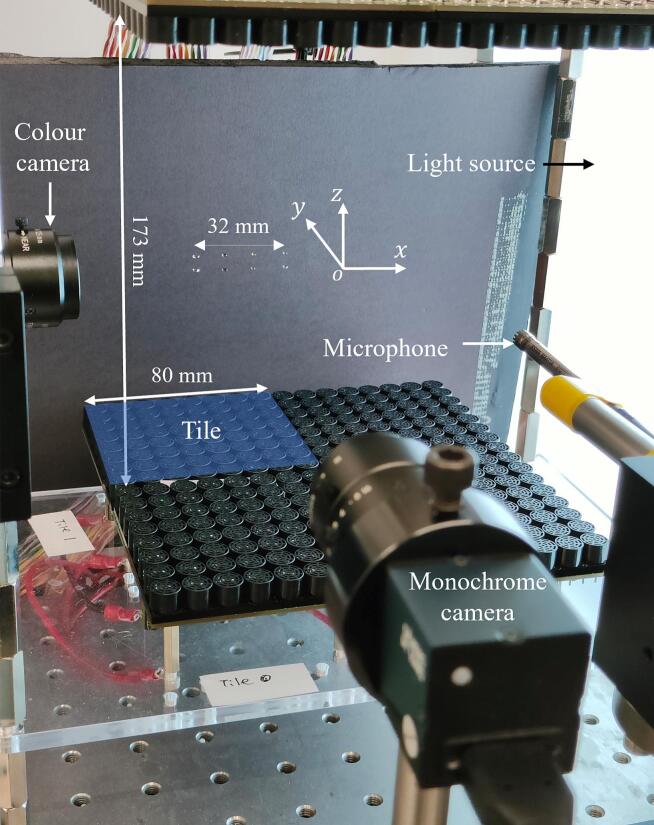
Phased arrays made of 10 mm 40 kHz transducers, cameras, and a microphone that was part of the acoustic scanning system with 4 columns of levitated droplets.

The phased arrays are the same as those described by the authors in [18] and shown in Fig. 1 with key geometrical and transducer parameters listed in Table S1. The control software has a graphical user interface shown in Fig. S1 and described in the SI. The acoustic scanning system (Fig. S2 and described in the SI) to measure the acoustic field had the following main components:

(1) a control board (Arduino Mega 2560)
(2) three translation stages (*x*, *y, z*) each connected to a driver (MC556C) that received pulse and direction signals from the control board and independently controlled movement in three directions
(3) an oscilloscope (Siglent SDS1104X-E 4Ch, Siglent Technologies Co., Ltd., Shenzhen, China) connected to the host capturing and displaying the microphone signal, which is then automatically saved by the program
(4) a microphone (MPA401 with preamplifier MA411A and ICCP MC102A; BSWA Technology Co., Ltd., Beijing, China) attached to the stage and connected with the oscilloscope
(5) a power supply (MODEL: GKF-200-24II, Powerld Enterprises Co., Ltd., Shenzhen, China) that provided 24 V DC voltage to power the three drivers.

The phase of the acoustic field was measured with respect to a reference signal fed to the second channel of the oscilloscope from the FPGA board.

A daylight wafer light box (model number: D/E/U/A 35040, 4 W LEDs) was used as a light source. Monochrome images were recorded using a DaHeng Imaging MER2000-19U3M-L USB3 camera (GeT Camera BV, Eindhoven, The Netherlands) with 5496 × 3672 pixels resolution. Colour images and movies were captured using a color camera (MER2-2000-19U3C, China) with 5496 × 3672 pixels resolution equipped with a widefield imaging 25 mm focal length lens (Navitar, USA).

### 2.3 Software and algorithm

**Programming of phased arrays:** The software reported by the authors in 2024 [18] was used to control the voltage and phases of the transducers (refer to Section S1 in the SI). The input phase files were entered step by step where the phases were calculated using our DS-PAT algorithm. This was useful for identifying the key steps of coalescence of droplets and analyzing the success or failure of merging.

**Acoustic scanning program:** As described in Section 1 in the SI, the entire scanning program encompasses both stage control and oscilloscope control. These two functions were realized through a host PC and a low-cost control board. For stage control a low-cost Arduino controller was used along with the associated development software. For oscilloscope control, National Instruments NI-VISA software was used.

**Calculation of acoustic fields:** Acoustic fields were calculated using the piston model of transducers [1] and using 4 different algorithms; Checkerboard, IBP, Diff-PAT and DS-PAT where DS-PAT was developed in this work and as discussed in Section 3 offered significant advantages compared to the other three algorithms. The DS-PAT algorithm was developed based on the hologram design method that relies on direct search [26,27]. As described in the SI, the DS-PAT is an optimization method that requires defining an objective function. In this work, two objective functions *C_1_(φ_ij_)* and *C_2_(φ_ij_)* were defined as follows:

(1) $$C_{1}\left(\varphi_{\mathrm{ij}}\right)=\sum_{n}\left|p_{n}-p_{n}^{\text{target}}\right|$$

Where *p_n_* and *p_n_^target^* were the calculated pressure and target pressure at a point with spatial coordinates *i*, *j* of transducers.

(2) $$C_{2}\left(\varphi_{\mathrm{ij}}\right)=-\sum_{n}\left|p_{n}\right|+\alpha \cdot \sqrt{\frac{\sum_{n}{\left|\left|p_{n}\right|-\overset{¯}{\left|p_{n}\right|}\right|}^{2}}{N}}$$

where the second term on the right side is the standard deviation of amplitude at target points, *N* and |*p_n_*| with an overline are the total number of target points and the average of calculated pressure at these points respectively, and *α* is a variable parameter. This objective function maximizes the magnitude of the pressure at the target points while maintaining uniformity and ignoring phase.

The DS-PAT was designed to find a global minimum in the parameter space of all the transducers’ phases. The phase of each transducer can lie between 0 and 2π discretized into 64 steps. Thus, in the initial state of the DS-PAT algorithm, transducers were either assigned a random phase from 0 to 2π×(63/64) or uniform phase of 2π×(63/64). For these initially set phases of transducers, the value of the objective function was calculated. For each transducer, the phase was varied, and the new value of the objective function was calculated and compared with the previously calculated value of the objective function. If Δ*C <* 0, the new phase was accepted, otherwise the new phase was accepted according to annealing principle (see Section S2 in the SI for details). Furthermore, simulated annealing was built in the DS-PAT but simulated annealing did not play a critical role in the optimization cases of this work (see Fig. S10).

### 2.4 Procedure

The optimization algorithm, experimental procedure and methods for data analysis are provided below.

• *Optimization of phases:* There were 2 cases in this work. One was parallel coalescence and the other sequential coalescence.
• For parallel coalescence, equal numbers of focal points were created at two columns with a separation distance (usually larger than 10 mm, otherwise droplets will directly merge) at the first step. Subsequently, a series of 2-column traps with smaller separation distance at each step was created. The separation distance between the two columns was reduced from the initial value to a final value of zero in a total of either 8 (sub-section 3.2.1) or 2 steps (sub-section 3.2.2).
• For sequential coalescence, there were 4 focal points, forming 4 columns of droplets. The first 7 steps were used to merge the left two pairs of droplets and the remaining 7 steps were used to merge the right two pairs of droplets. For all these steps, we used DS-PAT to calculate optimum phases for all transducers.
• Levitate, move and merge droplets: We used the developed software to send phase information to the transducers. EPS particles were used as markers for the trap positions. 2 µL droplets of 0.25 mM amaranth solutions were then dispensed at the trap positions and the EPS particles removed. When all the droplets were positioned, phase information was sent to merge droplets.
• *Chemical reactions:* We used sequential coalescence to conduct the Belousov–Zhabotinsky (BZ) oscillating reaction. Stock solutions of 0.5 M KBrO_3_, 1 M KBr, 1 M CH_2_(COOH)_2_ and 0.5 M Ce(NH_4_)_2_(NO3)_6_ were prepared in 0.9 M sulfuric acid. A stock solution of 0.5 % (w/v) ferroin was made by adding 0.024 g iron (II) sulphate heptahydrate and 0.046 g 1,10-phenanthroline in 10 mL 0.9 M sulfuric acid. For levitated droplets, solutions were made to obtain optimum concentrations in the merged droplets as shown in Table 1. Droplets of the following solutions were levitated: (1) mixture of KBrO_3_, KBr andCH_2_(COOH)_2_, (2) Ferroin, (3) Ce(NH_4_)_2_(NO3)_6_ and (4) H_2_SO_4_. 4, 2, 2 and 2 µL droplets of each solution, respectively were pipetted into 4 separate columns. These droplets were then merged using the designed sequence. The results of the coalescence process and following reactions are discussed in Sub-section 3.3.2.

**Table 1:** The concentration of all chemicals at different stages.

Chemical	H_2_SO4	KBrO_3_	KBr	CH_2_(COOH)_2_	Ferroin	Ce(NH_4_)_2_(NO_3_)_6_
Stock Concentration	0.9 M	500 mM	1000 mM	1000 mM	0.5 % (w/v)	500 mM
Dilute Concentration		192.5 mM	50 mM	250 mM	0.25 % (w/v)	40 mM
Final Concentration		77 mM	20 mM	100 mM	0.05 % (w/v)	8 mM
• *Scanning of acoustic field and data analysis:* The corresponding phase information with 10 V_PP_ voltage was sent to the phased arrays and the microphone was scanned in the *y* = 0 mm plane over an area of 30 mm × 60 mm with a resolution of 0.5 mm, which took ∼ 50 min. Fig. S3 shows the DOS window to initialize and start the scanning process. After all the data has been collected, a fast Fourier transform (FFT) for each time series was performed to obtain the corresponding amplitude and phase.
• *Data analysis for droplet images:* MATLAB was used to determine the area of liquid droplets and their centroids and extract grayscale values of red (R), green (G) and blue (B) channels of the colour camera.

## 3 Results and discussion

### 3.1 Comparison of algorithms

**Fig. 2:**
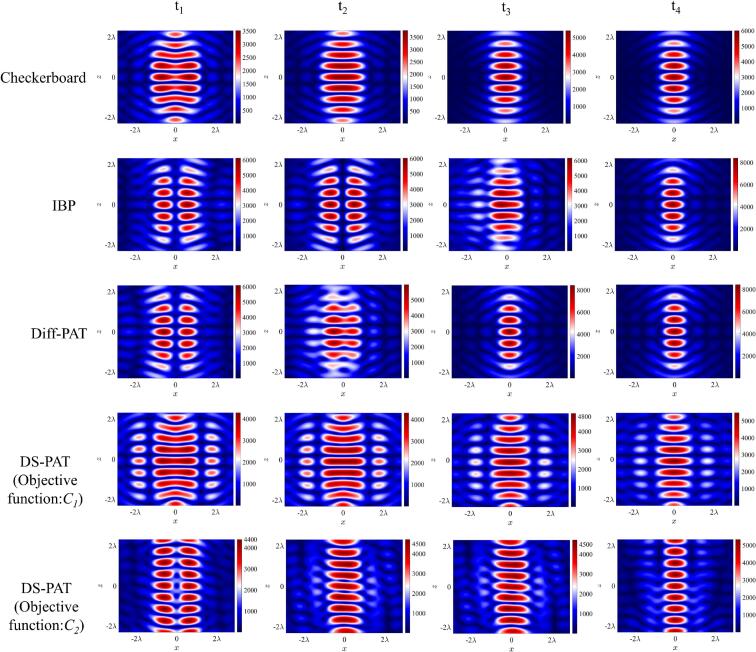
Simulation of the pressure field for different algorithms at different time steps. For the Checkerboard, IBP and Diff-PAT, the time steps correspond to 2-column target points separated by 10 mm (x: −5 mm, 5 mm), 8.8 mm (x: −4.4 mm, 4.4 mm), 7.0 mm (x: −3.5 mm, 3.5 mm) and 6.0 mm (x: −3.0 mm, 3.0 mm) respectively. For the DS-PAT algorithm (*C*_1_), the time steps correspond to 2-column target points separated by 10 mm (x: −5 mm, 5 mm), 9.6 mm (x: −4.8 mm, 4.8 mm), 9.2 mm (x: −4.6 mm, 4.6 mm) and 8.8 mm (x: −4.4 mm, 4.4 mm) respectively. For the DS-PAT algorithm (*C*_2_), t_1_ corresponds to 2-column target points separated by 10 mm (x: −5 mm, 5 mm), t_2_/ t_3_ and t_4_ correspond to several columns of target points distributed along the range of 8.0 mm (x: −4 mm to 4 mm with 1 mm interval) and 6.0 mm (x: −3 mm to 3 mm with 1 mm interval), respectively.

All 4 algorithms; Checkerboard, IBP, Diff-PAT and DS-PAT (2 kinds of objective functions) were used to calculate phases of transducers for coalescence of two columns of traps. As shown in Fig. 2, the two columns of traps were moved from a separation distance (*Δd*) of 10 mm to 6 mm. The pressure distributions obtained using different algorithms are shown in Fig. 2. Fig. 2 shows that Checkerboard algorithm resulted in a smooth merging of two columns of traps. Consequentially, the droplets can gradually approach each other and merge, allowing successful coalescence. In contrast, for IBP and Diff-PAT, the actual distance between two columns of traps stayed fixed when *Δd* < 10 mm, and when *Δd* becomes smaller than a certain distance, the two focal points suddenly merged into one with high impact. Correspondingly, the input voltages in the merged step should be reduced to a suitable value to prevent coalescence and atomization happening simultaneously. As shown in Videos S1-3, the change in input voltage from higher values to lower values avoided atomization. The voltage must not be too high after coalescence to prevent merged droplets from atomizing or shattering into smaller droplets. Equally, the voltage must not be too low otherwise droplets will fall before coalescence. However, this approach imposes a limitation on the maximum number of droplet pairs that can be merged simultaneously, because if the voltage is reduced to a low level, the droplets away from the central focal points with lower pressure will inevitably become unstable and fall. As a result, the most stable experimental case until this work was the coalescence of three pairs of droplets, which has already been successfully demonstrated by the authors [18].

As shown in Fig. 2, similar to the Checkerboard, the DS-PAT algorithm with objective function *C_1_(φ_ij_)* resulted in smooth, continuous changes in pressure fields for merging of columns of traps without having to change the input voltage as a function of time. In comparison, DS-PAT with objective function *C_2_(φ_ij_)* allowed creation of wide traps. In Fig. 2, at each selected z coordinate, wide traps were created by 7 focal points along the x direction each separated by 1 mm. The results between the optimization cases with simulated annealing and without simulated annealing are compared in Fig. S10. It shows that the two cases were similar, demonstrating that the simulated annealing did not play a critical role in the optimization cases of this work.

Other key points to note from Fig. 2 are that the maximum pressure produced by IBP was approximately 1.3 times higher than DS-PAT in the initial step and near 1.8 times higher in the merging step. Stable levitation was typically limited to 4 pairs, and the stable coalescence of droplets is ≤ 3 pairs with IBP. Video S2 shows one successful coalescence of 3 pairs of droplets with input voltage changed from 10 V_PP_ (initial step) to 8 V_PP_ (merging step) but also a failed coalescence with input voltage changed from 10 V_PP_ to 7 V_PP_. While IBP could sometimes stably levitate up to 4–5 pairs of droplets, its performance on levitation and coalescence of 4–5 pairs of droplets was quite unreliable. In contrast, as shown in Videos S4 and S5, two columns each containing 4 and 6 droplets were successfully merged using DS-PAT with objective functions *C_1_(φ_ij_)* and *C_2_(φ_ij_)*, respectively.

### 3.2 Parallel coalescence of multiple-pair droplets

Firstly, we showed that the DS-PAT algorithm can result in smooth, continuous changes in pressure fields when columns of traps are merged and hence allowed coalescence of 4 pairs of droplets. Secondly, DS-PAT was optimized to create uniform and wide traps for increasing the number of droplets that can be levitated in each column to 6 and for merging pairs of columns each containing 6 droplets, respectively.

#### 3.2.1 Continuous varying traps

Continuous varying traps mean that the pressure field changes smoothly and continuously as the two columns of traps are brought closer together and eventually merged. The continuous changes in pressure fields keep the pressure at a level that is enough to pull the droplets together but not too high to result in atomization of the droplets. Although smooth, continuous changes in pressure fields can be obtained via the Checkerboard algorithm, the DS-PAT is beneficial because it results in higher pressure values when the two columns are separate as shown in Fig. 2. Equally, DS-PAT is more versatile than Checkerboard because it can be used to create more than two columns of traps. To obtain continuous varying traps using the DS-PAT algorithm, the objective function *C_1_(φ_ij_)* was used. We set 12 focal points with 6 points per column in the plane at *y* = 0 mm. The z-coordinates of the focal points are provided in Section 3.2.1 in the SI. At target points, phases were optimized to achieve coherent wave interference, maximizing sound pressure. In regions away from the target points, significantly reduced pressure was observed. Hence, the influence of pressure in regions away from the target points was negligible. A comparison of Fig. S6 to S8 in the SI showed that the separation distance in the x-direction between experimentally obtained columns of traps and hence droplets was 15.45 mm, which was greater than the target value of 14 mm in the simulations. The separation distance in the z-direction between the droplets was approximately 5 mm in experiments versus 4.3 mm in simulations. Equally, a comparison of Fig. S6 and S7 showed that the magnitude of pressure in experiments was different to simulations, which may be a limitation of the DS-PAT algorithm. There were no distinguishable separate focal points when the *x*-direction separation between the columns of traps was < 10 mm, which can also be observed in the Checkerboard-generated acoustic fields (Fig. 2). Even though the simulated pressure field obtained via the DS-PAT algorithm appeared to be able to levitate and merge six pairs of droplets, experimentally only 4 pairs of droplets at *z* = 1.40 mm, −3.56 mm, −8.60 mm, −13.70 mm could be successfully merged.

**Fig. 3:**
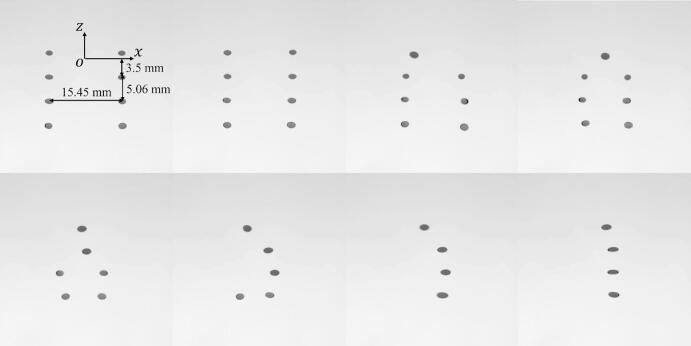
Images at each of the 8 steps used to merge 4 pairs of droplets one by one by moving the target separation distance between the columns of traps successively from 14.0 to 12.0, 10.0, 9.6, 9.4, 9.2, 9.0, and 8.8 mm.

The 8 steps used to merge four pairs of droplets using the DS-PAT algorithm with objective function *C_1_(φ_ij_)* are shown in Fig. 3. Repeated experiments indicated that the complete coalescence of four pairs of droplets usually needed five steps. As shown in Fig. 3, the upper droplets merged at first and lower droplets followed one by one. This sequential merging of droplets can be explained by considering Figs. S6 and S7, which show that the merging of the upper traps resulted in pressure profiles that allows the droplets to move “downhill” towards each other, facilitating merging. In contrast, the bottom trap at the same step would require the droplets to move “uphill” to merge.

#### 3.2.2 Uniform and wide traps to merge six pairs of droplets

While it was possible to create smooth, continuous pressure fields using the DS-PAT algorithm with *C_1_(φ_ij_)*, this objective function struggled to find solutions for large numbers of traps. This limitation arose because *C_1_(φ_ij_)* is based on complex pressures, which was very difficult to achieve for all focal points especially as the number of focal points increased. To address this challenge, another objective function *C_2_(φ_ij_)* was defined.

**Fig. 4:**
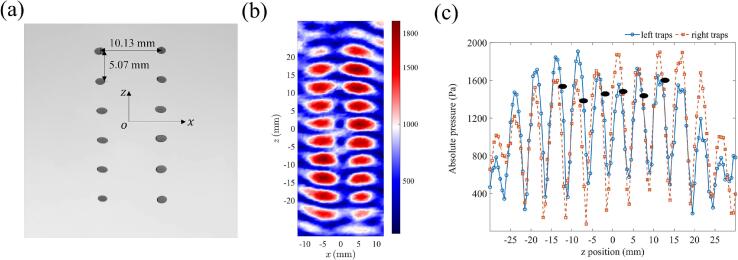
The initial step before coalescence. (a) 12 levitated droplets with 6 droplets in each column and the horizontal and vertical distances were in-line with the simulation results, (b) The scanned pressure contained two columns of traps with at least 7 complete antinodes per column, and (c) The extracted pressure along the left and right columns. The *z*-coordinates of the levitated droplets extracted from (a) are 12.20 mm, 7.18 mm, 2.15 mm, −2.81 mm, −7.70 mm, and −12.64 mm. These positions are marked by black ellipses in (c).

The objective function *C_2_(φ_ij_)* was based on the minus summation of all the absolute pressure plus the standard deviation of all the absolute pressures. As *C_2_(φ_ij_)* only considered pressure amplitudes, it enabled creation of large numbers of focal points with comparable pressures while maximizing the pressure at each focal point. As shown in Fig. 4 (a), the phase patterns generated using the DS-PAT algorithm with objective function *C_2_(φ_ij_)* allowed stable levitation of up to six pairs of droplets. This was made possible by the truly global nature of DS-PAT algorithm (as evidenced by Fig. S9 in the SI) and contrasts with existing algorithms that could not find the pressure field for levitating two columns with 6 droplets in each column.

Fig. 4 (a) shows that the lateral and axial distances between the droplets aligned well with the target configuration. However, the scanned pressure field (Fig. 4 (b)) revealed that the droplets are not precisely aligned with the nodes but positioned closer to the antinodes, as shown in Fig. 4 (c). This can be explained by considering that droplets also experience gravity, which is different from the phenomenon in microfluidics [31,32] where gravity can be ignored and only acoustic radiation force dominates. All droplets in Fig. 4 (a) have an ellipsoidal shape because they are confined much more strongly in the z- than in the x-direction as evident from the spatial pressure profile in Fig. 4 (b). Fig. 4 (a) highlights small differences in ovality of levitated droplets that are likely to be because of the differences in the spatial profile of different nodes as shown in Fig. 4 (b). The shape of acoustically levitated droplets can be determined by the Young-Laplace equation [[33], [34], [35]]. The deformation of droplets can be controlled by designing the pressure magnitude of the traps in the x-, y- and z-directions. The stronger the confinement in one direction compared to the other, the larger the deformation of droplets in that direction is, resulting in ellipsoid shaped droplets. Another factor that determines the deformation of droplets is the surface tension of the levitated materials. For example, we have observed that a few organic solvents with lower surface tension experiences larger deformation than de-ionized water. The droplets (shown as black ellipses in Fig. 4 (c)) levitated at similar z positions for the two columns.

**Fig. 5:**
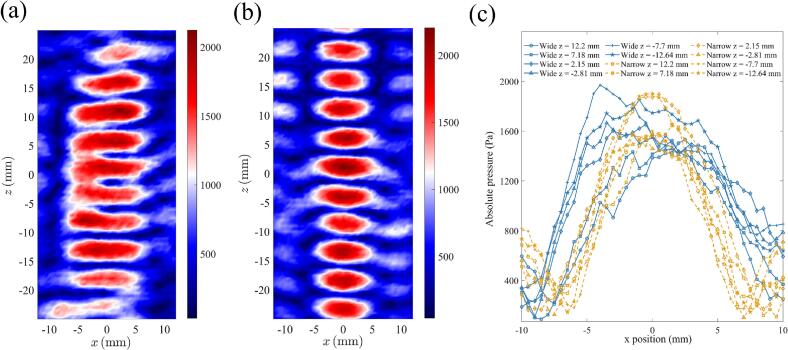
The pressure at the second step of the droplet merging process for (a) wide trap (showing regions where two focal points are connected at the edges along *z* = 2.15 mm), (b) narrow trap and (c) comparison of pressure profiles along the *x* direction for wide and narrow traps.

**Fig. 6:**
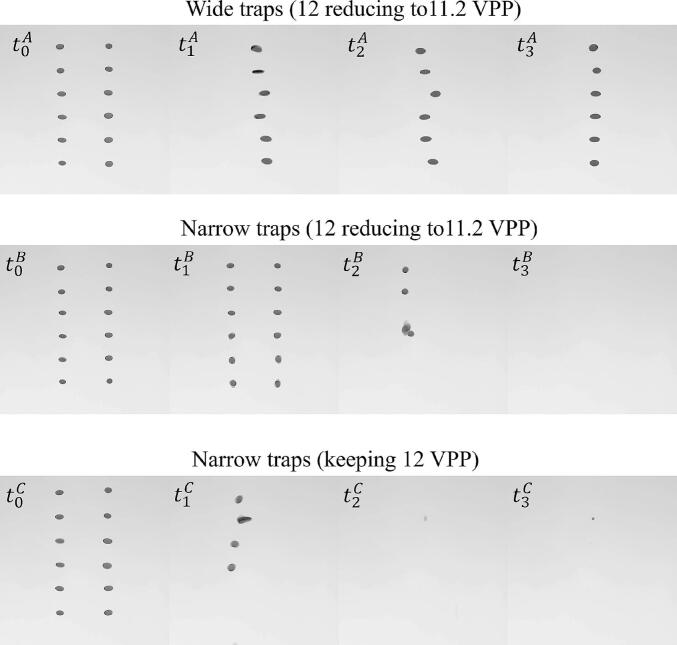
Comparison of coalescence of 6 pairs of droplets based on wide and narrow traps showing that overall, wide traps were more successful in merging 6 pairs of droplets than narrow traps.

The successful merging of columns of traps can be achieved by creating wide traps with acoustic energy expanded in the x-direction. To construct wide traps at each *z* coordinate (*z*_1_ = 15 mm to *z*_7_ = -15 mm with a 5-mm interval leading to 7 focal points along the axial direction), focal points were created at *x* -4, −3, −2, −1, 0, 1, 2, 3 and 4 mm. The DS-PAT with *C_2_(φ_ij_)* objective function was successful in finding a solution for the transducer phases needed to create wide traps. These phases were fed into the levitator and the resulting pressure profile measured using the acoustic scanning system is shown in Fig. 5 (a), confirming that wide traps with width 8 ∼ 10 mm were successfully created using the DS-PAT algorithm. Subsequently, narrow traps with widths of 6 ∼ 8 mm were designed using DS-PAT and the corresponding pressure profile is provided in Fig. 5 (b). A comparison of the lateral pressure distributions of wide and narrow traps is presented in Fig. 5 (c). It shows that horizontal pressure of the wide trap is, as expected, generally 1 ∼ 2 mm wider than that of the narrow trap. Furthermore, Fig. 6 (case A) shows that wide traps allowed stable coalescence of up to 6 pairs of droplets — an achievement that has not been reported in previous studies. Occasionally, droplets at *z* = 2.15 mm escaped after merging as a result of a weak lateral trap which was caused by two connected focal points at the edges along *z* = 2.15 mm as illustrated in Fig. 5 (a). Nevertheless, the system consistently achieved the stable coalescence of at least 5 pairs of droplets. Fig. 6 (cases B and C) also shows that in case of narrow traps, the droplets fell either before or after merging. After merging droplets using wide traps, they were changed to narrow traps to restrict the lateral position of the droplets as shown in Fig. 6 (t_3_^A^).

### 3.3 Sequential coalescence of 4 columns of droplets and oscillating reactions

As discussed before, sequential coalescence of more than two columns of droplets is essential for performing reactions requiring sequential addition of more than two reagents. Thus, to demonstrate applications of acoustically levitated droplets for performing chemical reactions requiring sequential addition of more than two reagents, DS-PAT was used to perform the sequential coalescence of 4 columns of droplets. Firstly, a symmetric coalescence of 4 columns of droplets along the *x* axis was designed as a demonstration. Subsequently, this method was used to perform the BZ oscillatory reaction [28], but with the merge position moved to the off-center region of the phased arrays such that the merge position is at the center of the colour camera used to monitor the reaction.

#### 3.3.1 Sequential coalescence of droplets

The initial demonstration of sequential coalescence was performed on 8 droplets in 4 columns which were merged in a sequence. The key parameters for designing sequential coalescence are the selection of target points and coefficients *α* in the second term of objective function *C_2_(φ_ij_)*. Table S2 gives the chosen target points and coefficient *α*, which can range from 0.2 to 10. The choice of the value for *α* depends on the selection of target points and is partly based on experience. The simulated acoustic field of the 14 steps involved in sequential merging of 4 columns of traps is shown in Fig. S11 with Fig. S12 confirming that 8 droplets in 4 columns were successfully coalesced sequentially.

#### 3.3.2 Oscillating reactions in the sequential coalescence mode

Oscillatory chemical reactions have applications ranging from analysis [36] to synthesis [37] and driving smart materials [38] and studying the origin of life [39]. The oscillatory BZ reaction was previously studied by the authors in the TinyLev acoustic levitation system [2] but the chemicals were added into the levitated droplets manually. Here we utilized sequential coalescence to automatically conduct the BZ reaction. Overall, the BZ reaction involves oxidation of malonic acid by bromate ions, and is summarized in equation (3):

(3) $${\text{5CH}}_{\text{2}}{\left(\text{COOH}\right)}_{\text{2}}{\;\text{+ 3BrO}}_{\text{3}}^{\;\text{-}\;}{\;\text{+ 3H}}^{+}\to \text{3BrCH}{\left(\text{COOH}\right)}_{\text{2}}{\;\text{+ 2HCOOH + 4CO}}_{\text{2}}{\;\text{+ 5H}}_{\text{2}}\text{O}$$

It is a nonlinear oscillating reaction system with several intermediates and reactions. If the system is added with a suitable metal ion, up to 10 intermediate reactions can exist in the system [2]. Here we introduced cerium (IV) ammonium nitrate (Ce(NH_4_)_2_(NO3)_6_) as the metal catalyst, resulting in the color of the BZ reaction to change between yellow and colorless. To make the color oscillations more prominent, ferroin (i.e., tris(1,10-phenanthroline) ferrous sulphate) can be added.

**Fig. 7:**
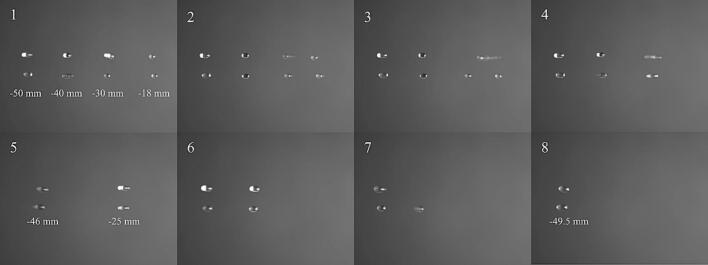
Selected frames of sequential coalescence (See video S7 for complete process). There were 4 columns initially. From left to right, the first column is 4 μL bromate/bromide/malonic acid; the second column is 2 μL Ferroin; the third column is 2 μL Ce(NH_4_)_2_(NO3)_6_ and the fourth column is 2 μL H_2_SO_4_. The third and fourth columns were merged at *x*  = -25 mm first and then the first and second columns were merged at *x*  = -46 mm. Finally, the remaining 2 columns of droplets were merged at *x*  = -49.5 mm. The upper and lower droplets merged with a slight time difference, so the reactions began at slightly different times.

The *x* coordinates of the 4 columns of droplets are provided in Fig. 7. The x-coordinate of the column of droplets obtained after sequential merging of the 4 columns was −49.5 mm, which aligned with the center of the colour camera. Firstly, we used DS-PAT to coalesce 2 μL of Ce(NH_4_)_2_(NO3)_6_ solution and 2 μL H_2_SO_4_. This resulted in Ce(NH_4_)_2_(NO3)_6_ droplets at *x*  = -25 mm with half the concentration to the original concentration (frame 4 in Fig. 7). Secondly, DS-PAT was used to merge 4 μL bromate/bromide/malonic acid solution and 2 μL Ferroin solution. The resulting droplets were located at *x*  = -46 mm (frame 5 in Fig. 7). This sequence was chosen because if the bromate/bromide/malonic acid and Ce(NH_4_)_2_(NO3)_6_ would have merged first, the chemical reactions would be activated very quickly. After 4 columns were merged into 2 columns, we switched to the Checkerboard method to rapidly generate the phases needed to move the final 2 columns of droplets closer and coalesce them (frame 8 in Fig. 7).

**Fig. 8:**
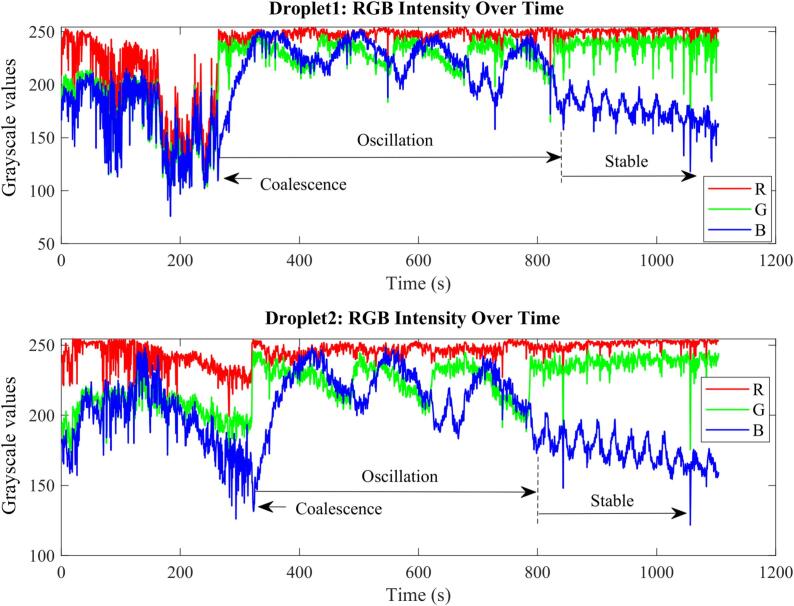
Grayscale values of red (R), green (G) and blue (B) channels of the colour camera over time. The intensity value is averaged over the two fixed rectangles shown in Fig. S13. The grayscale value of the red channel stayed the same while large oscillations in the grayscale values of the green and blue channel were observed. It also shows that Droplet 2 has one less oscillation than Droplet 1. (For interpretation of the references to color in this figure legend, the reader is referred to the web version of this article.)

After all the droplets were merged, the BZ reaction started and showed the oscillating color change (see Fig. S13). In Fig. 8, both green and blue channels of the colour camera showed strong oscillations while the red channel showed very little change, which is consistent with Songsaeng *et. al.* [2]. Based on the blue and green traces in Fig. 8, while droplet 1 experienced 4 oscillations, droplet 2 experienced only 3 oscillations. The experiments were repeated and, in all experiments, droplet 1 showed a higher number of oscillations than droplet 2 (See the explanation in S3.3.2. It may be caused by the distribution of acoustic field in Fig. S14). Furthermore, the number of oscillation cycles was less than reported by Songsaeng *et. al.* [2], which showed more than 10 cycles. This may be caused by the duration between the first and third coalescence was long, which might have resulted in the reactants to be partially consumed.

The blue channel showed the largest oscillation and hence was used to do the time course analysis by FFT. The FFT results showed that the frequency of oscillating reaction was 0.586 min^−1^ for both droplets 1 and 2. This is lower than 0.702 min^−1^ reported in Songsaeng *et. al.* [2]. This may be because TinyLev produces higher pressure and hence stirs and heats the droplets more, resulting in a faster reaction. As stated previously, the shape of levitated droplets in this work was ellipsoid, which have a different surface area to volume ratio than for example, spherical droplets. The surface to volume ratio of droplets influences interfacial processes and evaporation [35], which in turn affects the rate of chemical reactions. As discussed, the shape of droplets is determined by the spatial distribution of acoustic pressure. In future, the spatial distribution of acoustic pressure will be tailored to study the relationship between the shape of droplets and the rate of chemical reactions.

## 4 Conclusions

The coalescence of levitated droplets in an acoustic wave field is a complex dynamic process. A new algorithm, DS-PAT, for generating phase information for arrays of ultrasonic transducers has been developed and used to generate more pairs of traps than possible with previous algorithms (Checkerboard, IBP and Diff-PAT). This was achievable because of DS-PAT’s ability to find a global minimum. Two objective functions were defined for DS-PAT. The first objective function, which used both the amplitude and phase of pressure, was able to generate smooth, continuously varying pressure fields similar to Checkerboard for successful coalescence of up to 4 pairs of columns of droplets. The second objective function, which only used the amplitude of pressure, allowed generation of focal points with similar pressure to levitate 6 pairs of droplets in two columns. Equally, the second objective function allowed generation of wide traps that were shown to be suited for coalescence of 6 pairs of droplets in two columns, which has never been achieved by previous algorithms including Checkerboard, IBP and Diff-PAT. Subsequently, we successfully achieved the sequential coalescence of 4 columns of droplets to implement reactions that require sequential addition of more than 2 reagents in levitated droplets. The sequential coalescence of 4 columns of droplets was applied to start and then study an oscillating reaction in levitated droplets. Pressure intensity causing local vibrations, streaming and heating the environment may further influence chemical reactions in levitated droplets. Future work will investigate these effects, as well as the maximum number of droplets that can be levitated and merged with a fixed size array of transducers. Finally, ways of speeding up the DS-PAT algorithm will be investigated. Our research is a foundation of contactless chemical reactions. In future, the findings reported here can be applied to a diverse range of reactions including those that are strongly coupled to ultrasound.

## CRediT authorship contribution statement

**Jianqing Li:** Writing – review & editing, Writing – original draft, Visualization, Validation, Methodology, Investigation, Formal analysis, Data curation. **Nicholas J. Goddard:** Writing – review & editing, Validation, Software, Formal analysis, Data curation, Conceptualization. **Ruamsiri Songsaeng:** Writing – review & editing, Investigation, Data curation. **Ruchi Gupta:** Writing – review & editing, Visualization, Validation, Supervision, Resources, Project administration, Methodology, Funding acquisition, Formal analysis, Data curation, Conceptualization.

## Declaration of competing interest

The authors declare that they have no known competing financial interests or personal relationships that could have appeared to influence the work reported in this paper.
