# Supplementary material for: Coalescence of multiple pairs of levitated droplets using dual-side phased arrays
Source: Ultrason Sonochem. 2025 Mar 31;116:107327. doi: 10.1016/j.ultsonch.2025.107327 (PMC11999590; doi:10.1016/j.ultsonch.2025.107327)
Supplement: Supplementary Data 9 [file mmc9.docx]

**Coalescence of Multiple Pairs of Levitated Droplets Using Dual-Side Phased Arrays**

Jianqing Li^[[1]](#footnote-1)^, Nicholas J. Goddard^[[2]](#footnote-2)^, Ruamsiri Songsaeng^a^, and Ruchi Gupta^*a^

# S1 Software and instruments

**Software for phased arrays:** The graphical user interface (GUI, see Fig. S1) was used to manually or programmatically control the phases. For manual control of the phases, the algorithm was set to ‘None’, a csv file containing the phases of all the transducers was loaded, and ‘Send this step’ command was then clicked to send voltage and phases information to the transducers forming the phased arrays. As described in section 3.2.2 in the main article, we used DS-PAT and Checkerboard to realize the sequential coalescence. In Fig. S1 ‘Algorithm’ box, we used ‘None’ mode to load phases calculated using DS-PAT for coalescence of four columns into two columns of droplets. Subsequently, we switched to ‘Checkerboard’ mode and modified the focal positions in the input file to calculate the phases required for the third coalescence.


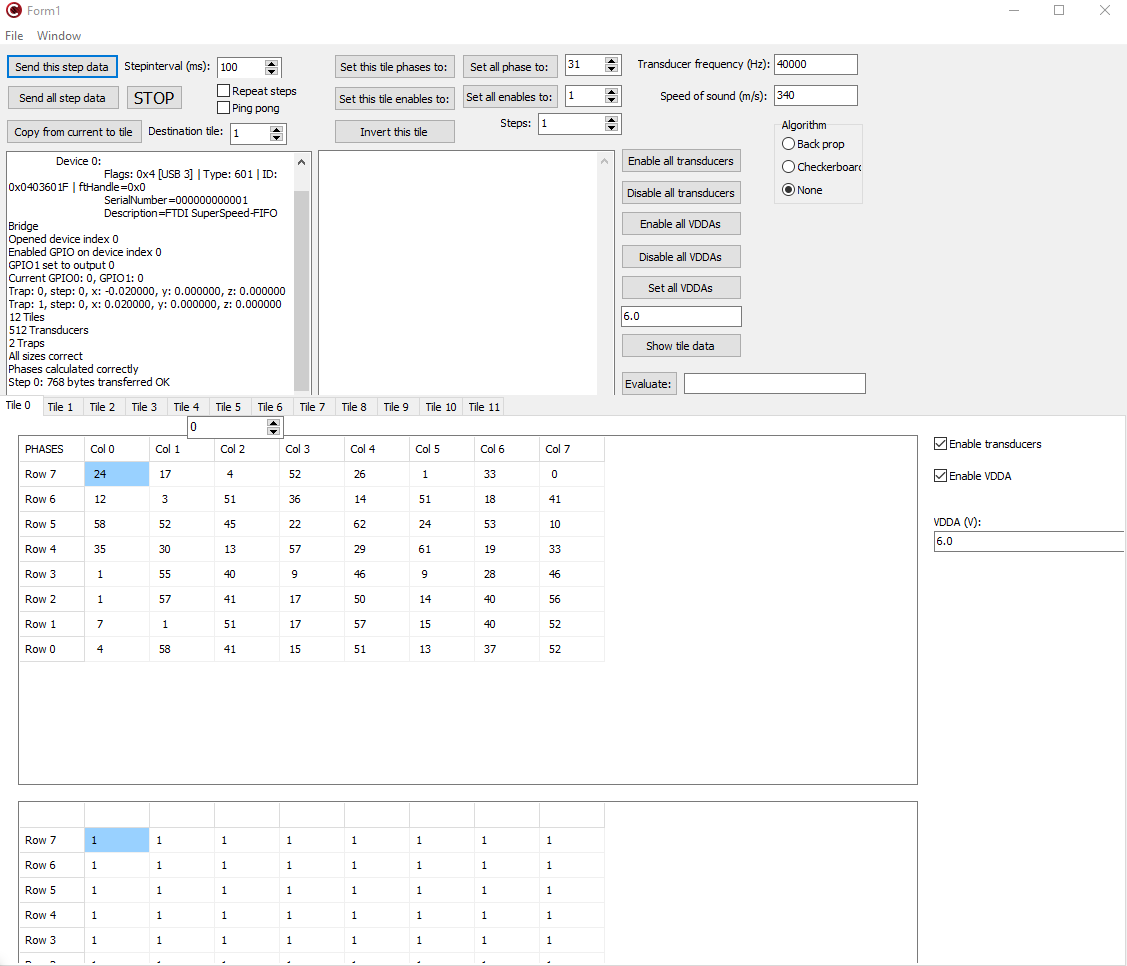


**Fig. S1: Software interface for sending signals to the transducers.**

**Configuration for acoustic scanning program:** we use the host to communicate with the Arduino control board. To set up the runtime environment, both the Arduino IDE (Integrated Development Environment for Arduino), which is a software application for writing, compiling, and uploading codes to Arduino boards, and Arduino for Visual Studio (a plug-in for Visual Studio 2022 that facilitates building, uploading, and debugging in Visual Studio) needs to be installed. For oscilloscope control, NI-VISA must be installed firstly. NI-VISA is a software framework developed by National Instruments (NI) that allow communication with various instruments and devices, providing the necessary interface for communication between host and the oscilloscope.

**Process of scanning and data processing:** The different components of the scanning system are shown in Fig. S2. The microphone was attached to the translation stage with translation freedom in all three spatial directions. The position of the translation stage and hence the microphone was changed with time. For each position, the microphone’s output was sent to the oscilloscope, and the data was saved as time series by running the program automatically using the DOS window shown in Fig. S3.


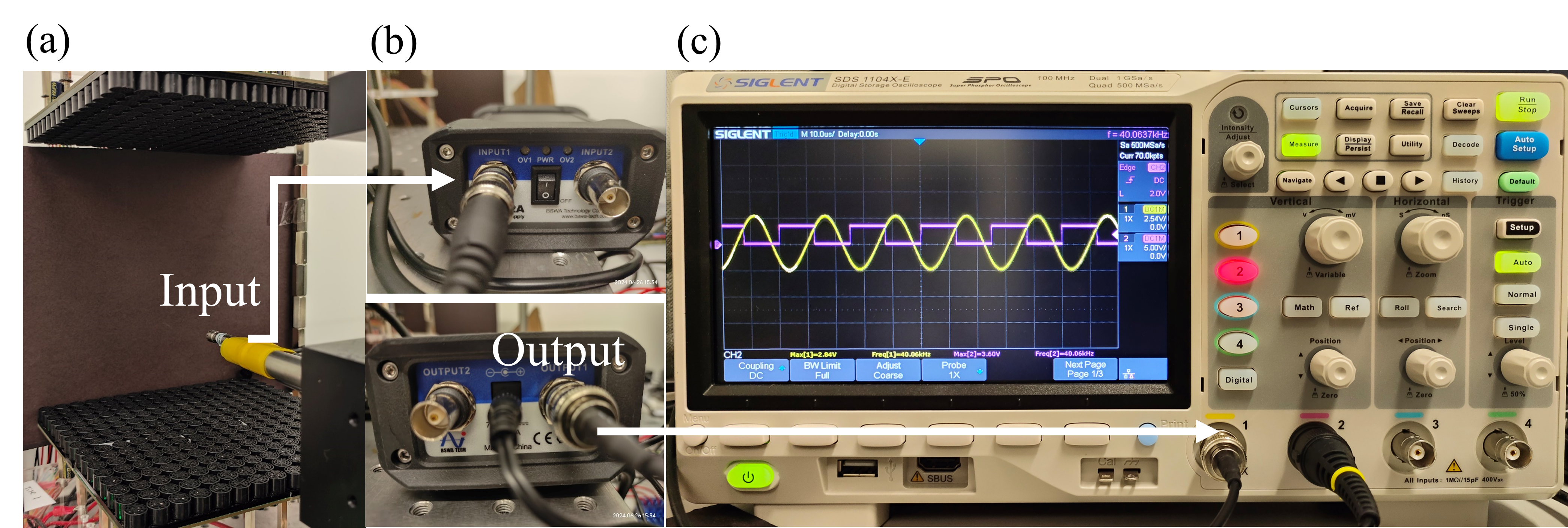


Fig. S2: A picture showing different components of the acoustic scanning system with (a) a microphone and preamplifier attached to the z translation stage, (b) ports that powered the microphone, received signal from microphone and transmitted signal to an oscilloscope, (c) the oscilloscope showing the received signal and transmitted the data to the computer for further analysis. There are two signals shown on the display of the oscilloscope where the yellow-coloured sinusoidal wave is the acoustic signal and the purple-coloured square waveform is the reference signal from the FPGA board.


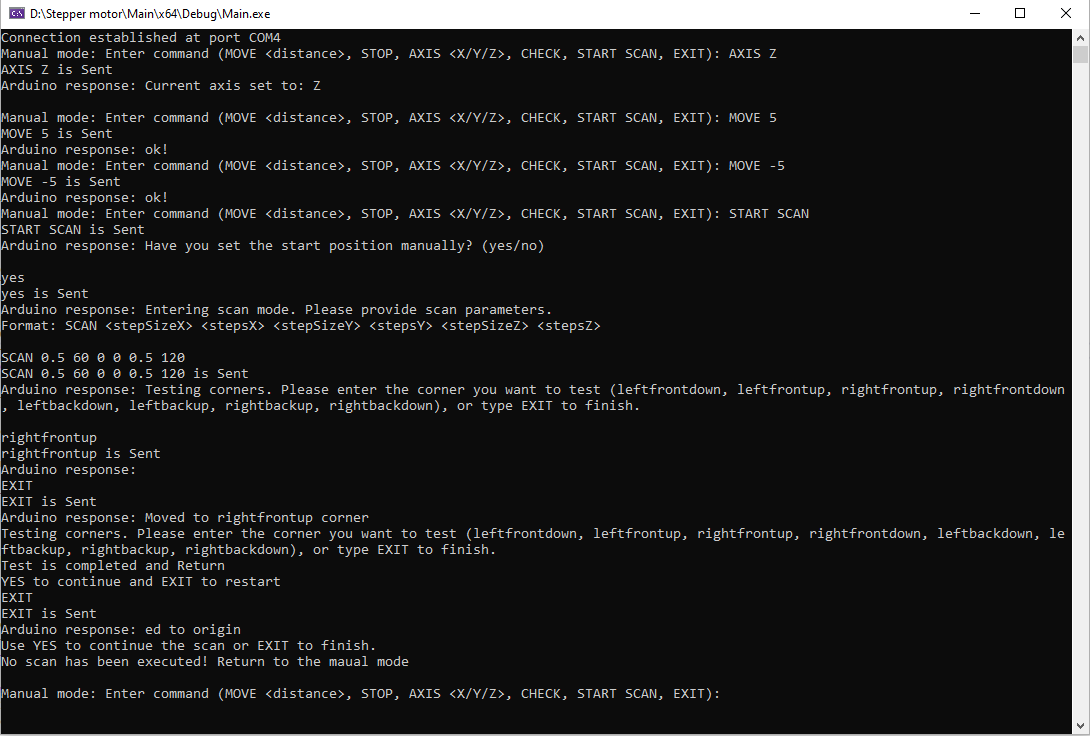


Fig. S3: A DOS window showing the commands used to interact with the acoustic scanning system.

The file system of the scanning program was divided into 3 parts including the main control program, the Arduino control program and the oscilloscope program shown in Fig. S4. All these files were already configured in the Visual Studio Integrated Development Environment. The ‘Main.sln’ file was opened and ran using the local windows debugger. The interaction with program was *via* the DOS window shown in the Fig. S3. We used the ‘AXIS’ and ‘MOVE’ command to adjust the start position of the microphone. We then used the ‘SCAN <stepSizeX> <stepsX> < stepSizeY> < stepsY> < stepSizeZ> < stepsZ>’ command to set the scanning steps and step length (usually 0.5 mm) along each direction. Typically, we used 61 × 121 steps (*x*🞨*z*) to cover an area of 30 mm × 60 mm with a resolution of 0.5 mm, taking approximately 50 minutes to do the entire scan. Then the ‘Testing corners’ mode was used to move the microphone to selected corners to validate that the coordinates of the scanned area are correct. Subsequently, further scans can be performed by entering ‘YES’ or the process can be ended by entering ‘EXIT’. After all the data has been collected, FFT was performed on each time series data to obtain the amplitude and phase. The difference between the phase determined via FFT and the reference signal (obtained from FPGA) was used to determine the phase of the pressure.


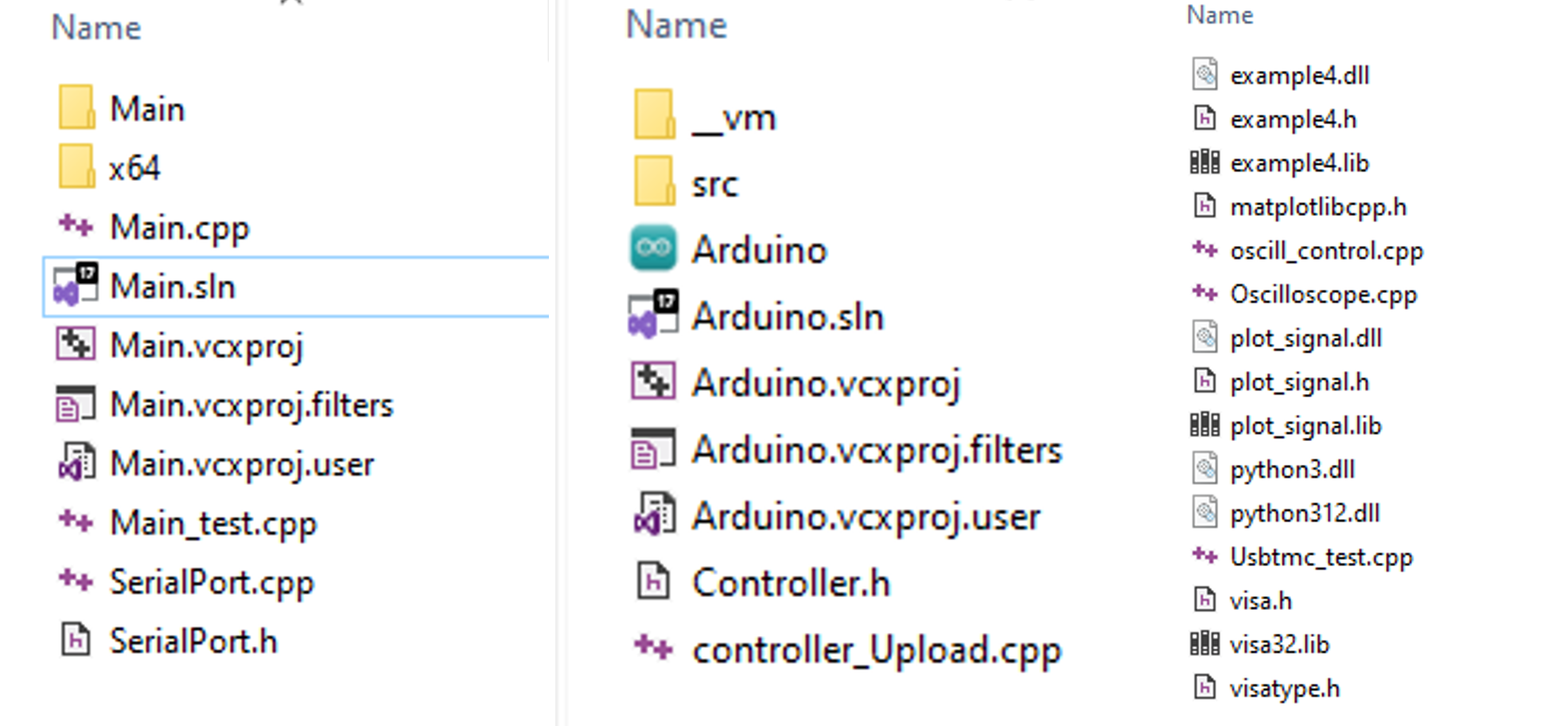


Fig. S4: The file systems of the acoustic scanning program. From left to right are the Main control program, Arduino control program (for controlling the movement of stages) and oscilloscope control program (for controlling the signal sampling).

# S2 DS-PAT algorithm

The working principle of the DS-PAT algorithm developed in this work is illustrated in Fig. S5.


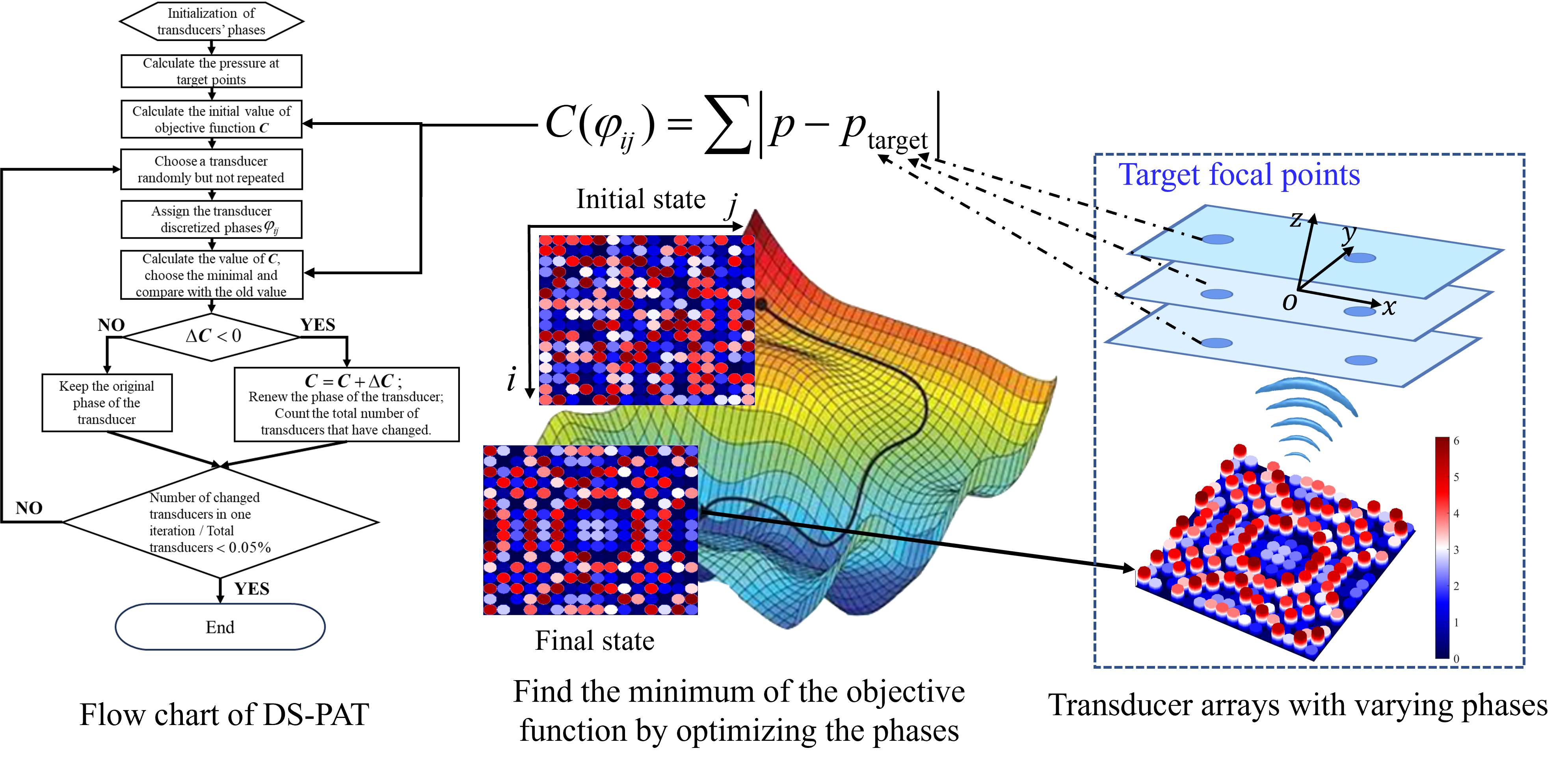


**Fig. S5: An illustration of DS-PAT algorithm that searches for phase combinations to minimize the objective function *C(φ_ij_)***

We defined two objective functions that are defined in the main manuscript. The parameters that we used for DS-PAT are listed in Table. S1. Some of these parameters were also used for the Checkerboard, IBP, and Diff-PAT algorithms. In Table S1, the end criterion is defined as the percentage of transducers that changed phases in one iteration divided by the total number of transducers. At the start of the optimization algorithm, values for parameters such as *α* were set based on experience. For example, generally speaking, when *α* was set to a high value, the pressure was more uniformly distributed over a wider distance but with low amplitude. The inverse was true if *α* was set to a low value.

Table S1: Parameters used in the DS-PAT

| **Number of transducers** | **Distance between top and bottom faces of the phased arrays (mm)** |  | **Diameter of transducers (mm)** | **Source velocity (m/s)** | **Sound velocity (m/s)** |
| --- | --- | --- | --- | --- | --- |
| Top face: 16×16  Bottom face: 16×16 | 173 |  | 10 | 0.795 | 346 |
| **Frequency (Hz)** | **Discretized levels of phases** |  | **Target amplitude (Pa)** | **Coefficient *α* in *C_2_(φ_ij_)*** | **Initial annealing temperature** |
| 40000 | 64 |  | 4000-6000 | 0-1000 | 10-100 |
| **End criterion** |  |  |  |  |  |
| 0.05% |  |  |  |  |  |

We also introduced the simulated annealing in DS-PAT which means the there is an initial temperature that will gradually decrease to zero with speed *T_k_* ~ *C*⋅0.9*^k^*, *k* is the number of iterations. When the temperature is high, it is easy to get out of the trap of local minimum. As the number of iterations increased, the probability of accepting an inferior solution is reduced. This is known as the famous Metropolis criterion:

 (S1)

# S3 Results

## S3.1 Comparison of algorithms

The ability of different algorithms to coalesce two columns of droplets was investigated. For this purpose, the parameters listed in Table S1 were used. Our findings for different algorithms are summarized below.

- **Checkerboard:** This algorithm resulted in continuous pressure distribution during the merging process. This is very helpful in making sure the stable coalescence of droplets. Video S1 shows the coalescence of 2 pairs of droplets using the Checkerboard algorithm.
- **IBP:** Video S2 shows merging of droplets using the IBP algorithm. The initial voltage was 10V p-p, reducing to either 7 or 8 V p-p as the droplets merged. At 7 V p-p the droplets merged but immediately fell out of the traps, while at 8 V p-p the merged droplets remained levitated. Fig. S5 shows that for IBP, the merging of the traps is not quite symmetric. Thus, it is likely that one column of droplets experienced stronger force towards the final position and the resulting collision at 7 V p-p knocked the merged droplets out of the trap.
- **Diff-PAT:** Video S3 shows merging of the droplets using the Diff-PAT algorithm.
- **DS-PAT:** Videos S4 and S5 show merging of the droplets using the DS-PAT algorithm with transfer function set to *C_1_(φ_ij_)* and *C_2_(φ_ij_)*, respectively.

## S3.2 Parallel coalescence using DS-PAT

**S3.2.1 Continuous varying traps**

The initial lateral target *x* coordinates of the two columns were *x*_1_^left^ = -7 mm, *x*_1_^right^ = 7 mm. The *z* coordinates of focal points along the axial line were *z*_1_ = 10 mm, *z*_2_ = 5.7 mm, *z*_3_ = 1.4 mm, *z*_4_ = -3 mm, *z*_5_ = -7.3 mm, *z*_6_ = -11.6 mm. The *z* coordinates of all focal points remained fixed in every step while the *x* coordinates of the two columns were changed as follows: *x*_1_^left^ = -7 mm, *x*_1_^right^ = 7 mm; *x*_2_^left^ = -6 mm, *x*_2_^right^ = 6 mm; *x*_3_^left^ = -5 mm, *x*_3_^right^ = 5 mm; *x*_4_^left^ = -4.8 mm, *x*_4_^right^ = 4.8 mm; *x*_5_^left^ = -4.7 mm, *x*_5_^right^ = 4.7 mm; *x*_6_^left^ = -4.6 mm, *x*_6_^right^ = 4.6 mm; *x*_7_^left^ = -4.5 mm, *x*_7_^right^ = 4.5 mm; *x*_8_^left^ = -4.4 mm, *x*_8_^right^ = 4.4 mm.


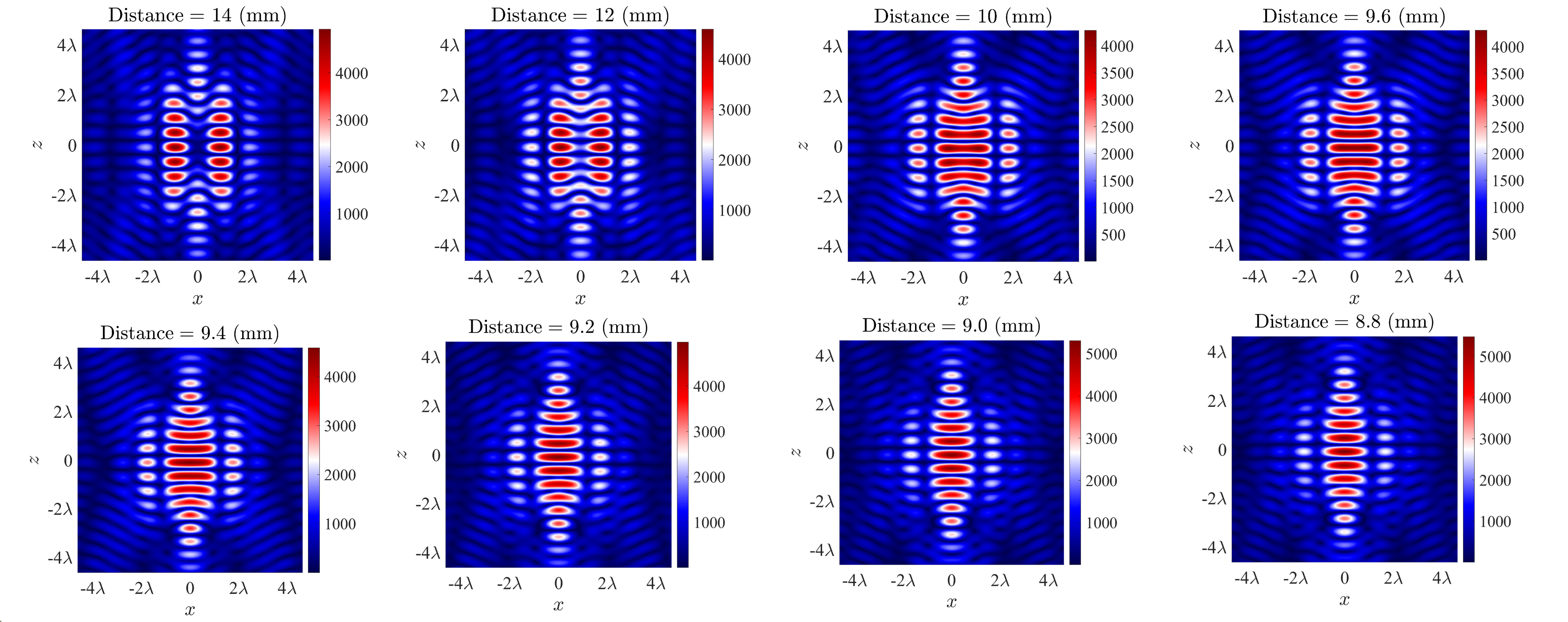


Fig. S6: Simulated pressure of the 8 steps used to merge two columns of traps with the objective function set to *C_1_(φ_ij_)*.

We scanned the pressure field with 30🞨40 mm^2^ area and noted that the pressure was not symmetrical around the *z* = 0 line (see Fig. S7). This may be because some of the transducers forming the top face of the acoustic levitator had failed. It is also because of this reason that droplets located above *z* = 5.5 mm were much harder to coalesce. Thus, we primarily limited the coalescence experiments to four pairs of droplets.


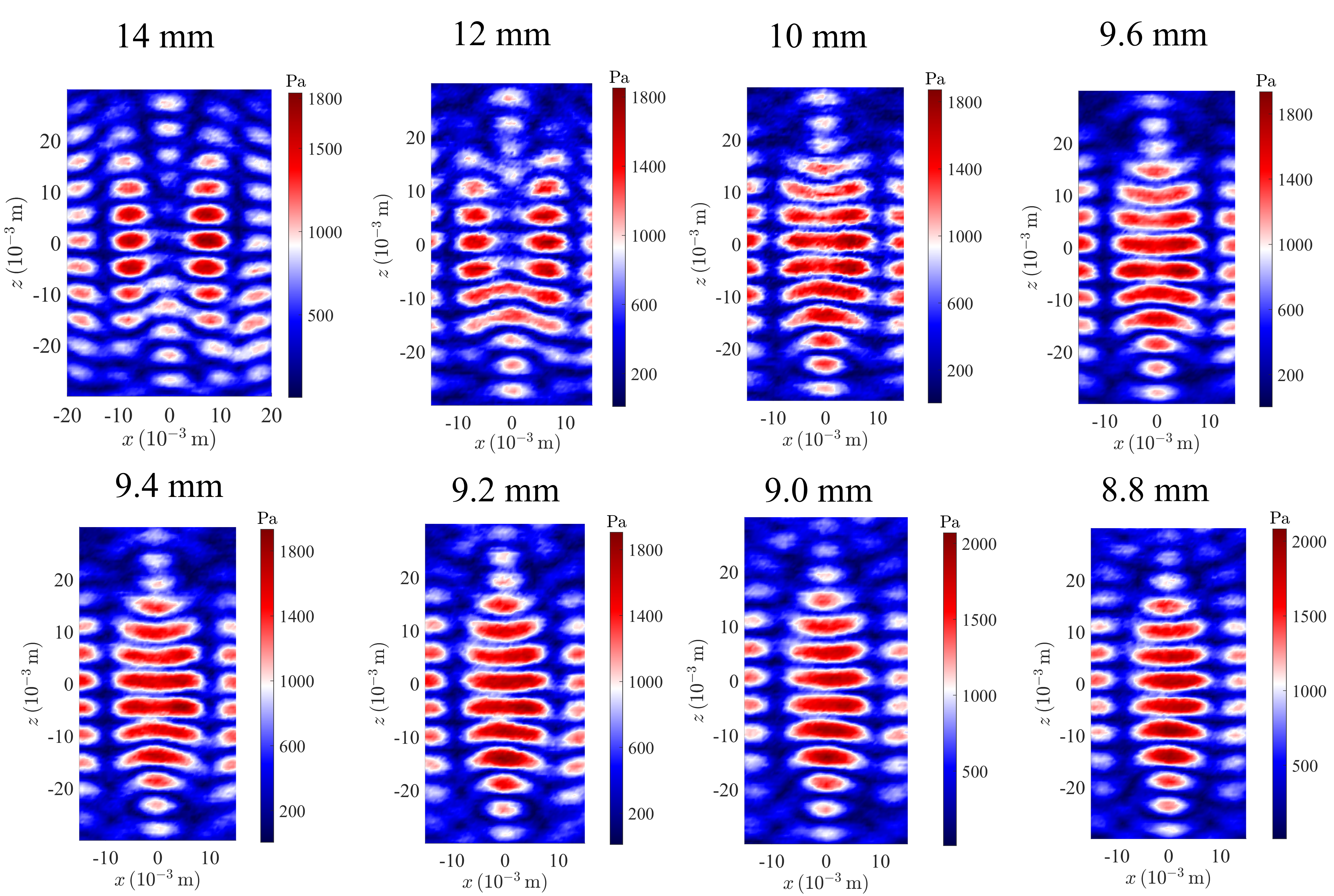


Fig. S7: Scanned pressure field at 8 steps to merge 4-pair droplets where the target separation distance in x-direction between two columns of traps was varied from 14 mm to 8.8 mm. When the distance was smaller than 9.4 mm, the 2 two columns of traps merged into one wide column.


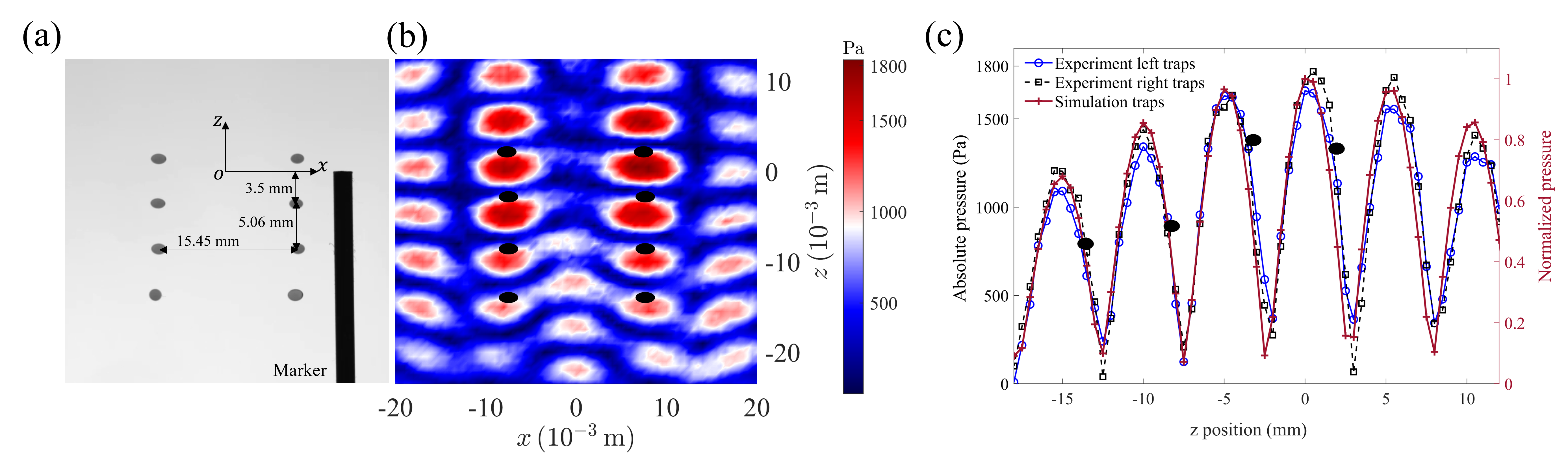


Fig. S8: (a) An image of the levitated droplets (2 μL 0.25 mM Amaranth solution), (b) scanned pressure distribution of the initial step in *y* = 0 and black ellipses indicate the approximate positions of levitated droplets, and (c) the extracted normalized simulated and experimentally obtained pressure profiles along the left and right columns with black ellipses indicating the approximate positions of levitated droplets.

**S3.2.2 Multiple nodes and wide focal pressure mode to merge 6 pairs of droplets**

Based on the phases of IBP, DS-PAT can still adjust the phase pattern to achieve lower value of *C*_2_ shown in Fig. S9, and a more ideal pressure distribution at the same time which is a direct proof of its global nature.


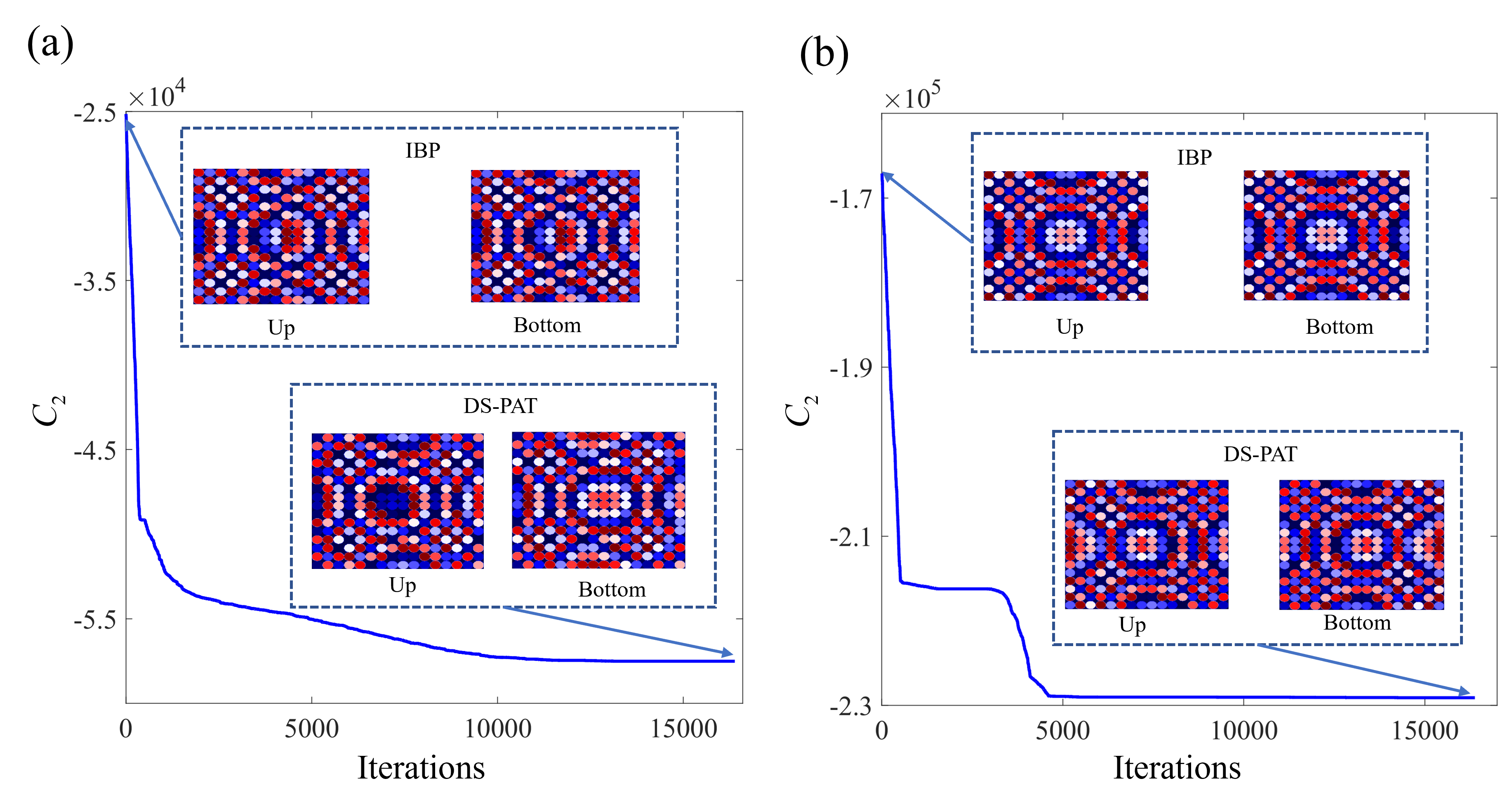


Fig. S9: Value of *C*_2_ versus iterations of the optimization process of DS-PAT for two steps in Fig. 4 and Fig. 5 of main text. In each step, the initial phases were generated by IBP but *C*_2_ can still decrease to find a better solution, which was made possible by the global nature of DS-PAT. (a) corresponds to *x*: -5, 5 mm in Fig. 4 and (b) corresponds to *x*: -4, -3, -2, -1, 0, 1, 2, 3, 4 mm in Fig. 5 respectively.


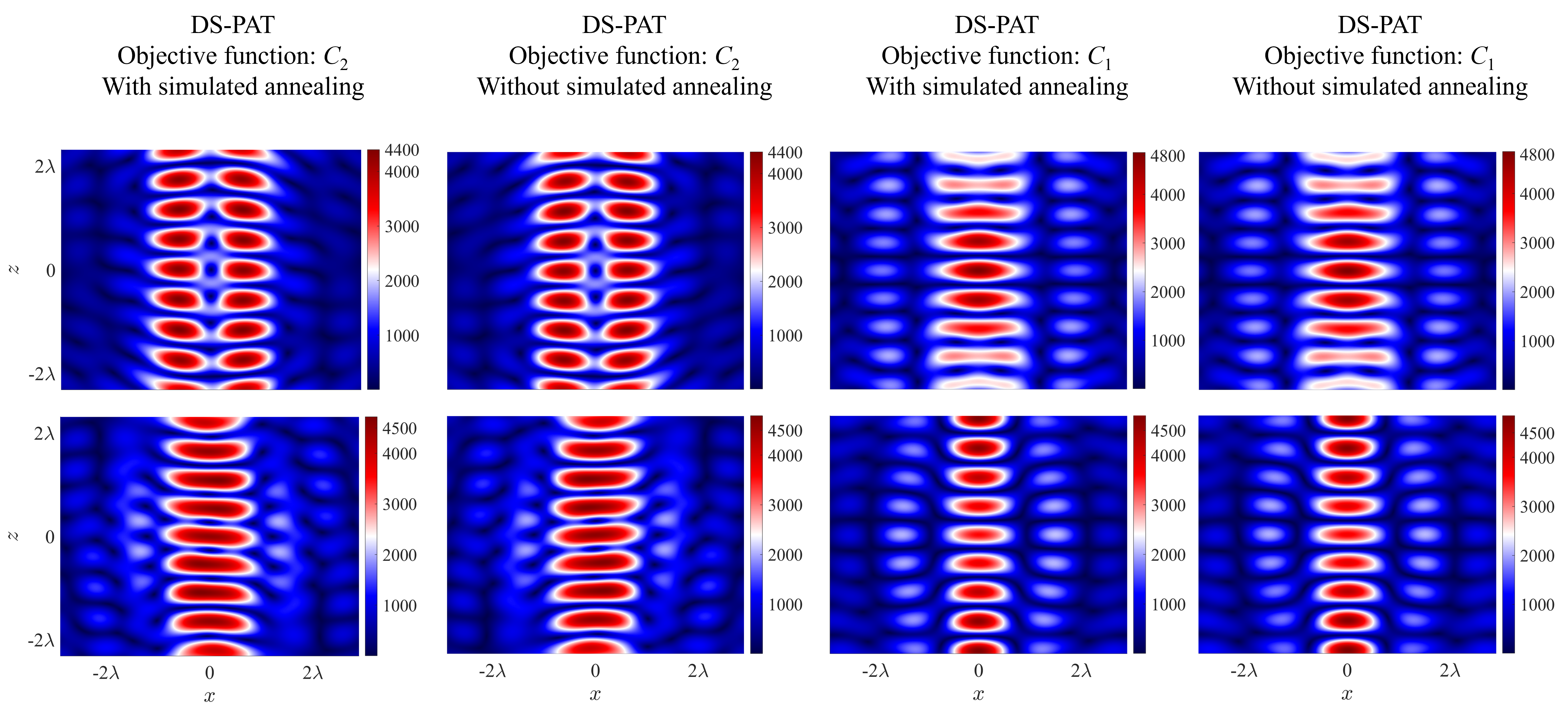


Fig. S10: Comparison of amplitude of the acoustic field for DS-PAT (*C*_1_ and *C*_2_ objective functions) with and without simulated annealing. The first row is the status that two columns are separate with *x* coordinates: -5, 5 mm; the second row is the status that two columns merge into one column where the target foci are set at *x*: -4, -3, -2, -1, 0, 1, 2, 3, 4 mm.

## S3.3 Sequential coalescence

**S3.3.1 Four columns merged into two columns**

We used objective function *C_2_(φ_ij_)*. The most challenging aspect of the sequential coalescence was selecting the focal coordinates especially the *x* coordinates and the coefficient *α* in the objective function. Fig. S11 and Table S2 highlights that the number of focal points were different in different steps. This is because when the target separation distance between the two left traps was 6 mm, the two focal points along the same *z* coordinate merged into one wide trap. Hence, for separation distances <6 mm, one target point was enough for the left traps and 3 target points in total. However, in the final 14^th^ step, we used 4 target focal points to construct wide traps that in our experience allowed successful coalescence of droplets much more repeatedly. As of now, the values for *α* were selected based on experience rather than established rules.

Table. S2: Parameters of optimization used in sequential coalescence

| **Step** | ***x*** | ***α*** | **Step** | ***x*** | ***α*** | **Step** | ***x*** | ***α*** |
| --- | --- | --- | --- | --- | --- | --- | --- | --- |
| **1** | (-20, -7, 7, 20) | 4 | **6** | (-20, -12, 7, 20) | 4 | **11** | (-17, 10, 20) | 0.5 |
| **2** | (-20, -8, 7, 20) | 4 | **7** | (-20, -14, 7, 20) | 1 | **12** | (-17, 11, 20) | 0.5 |
| **3** | (-20, -9, 7, 20) | 4 | **8** | (-17, 7, 20) | 0.2 | **13** | (-17, 12, 20) | 1 |
| **4** | (-20, -10, 7, 20) | 4 | **9** | (-17, 8, 20) | 0.5 | **14** | (-20, -14, 12, 20) | 10 |
| **5** | (-20, -11, 7, 20) | 4 | **10** | (-17, 9, 20) | 0.5 |  | | |

**
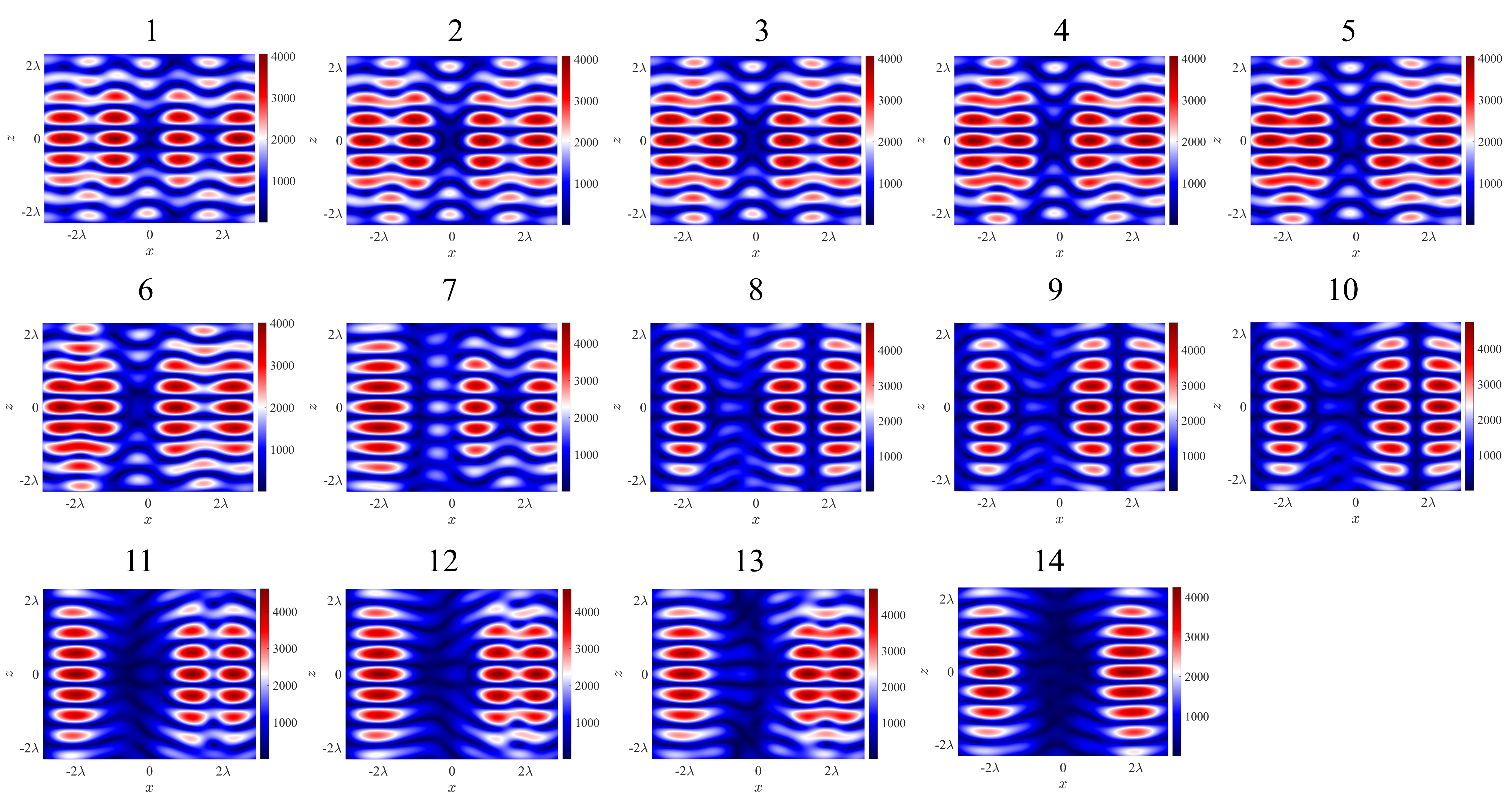
**

Fig. S11: Pressure at all steps used for sequential coalescence of four columns of droplets. The first 7 steps are for merging pairs of columns on the left and the remaining 7 steps for merging the columns on the right.


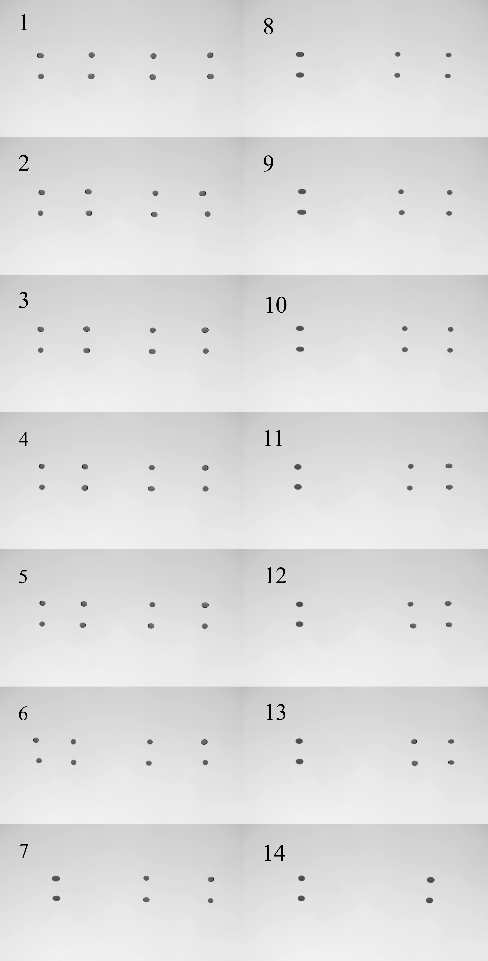


Fig. S12: All the 14 steps of sequential coalescence of droplets in 4 pairs of columns (2 μL 0.25 mM Amaranth solution) sequentially. The left 2 pairs of droplets are first merged and the right 2 pairs of droplets followed.

**S3.3.2 Sequential coalescence for oscillating reactions**


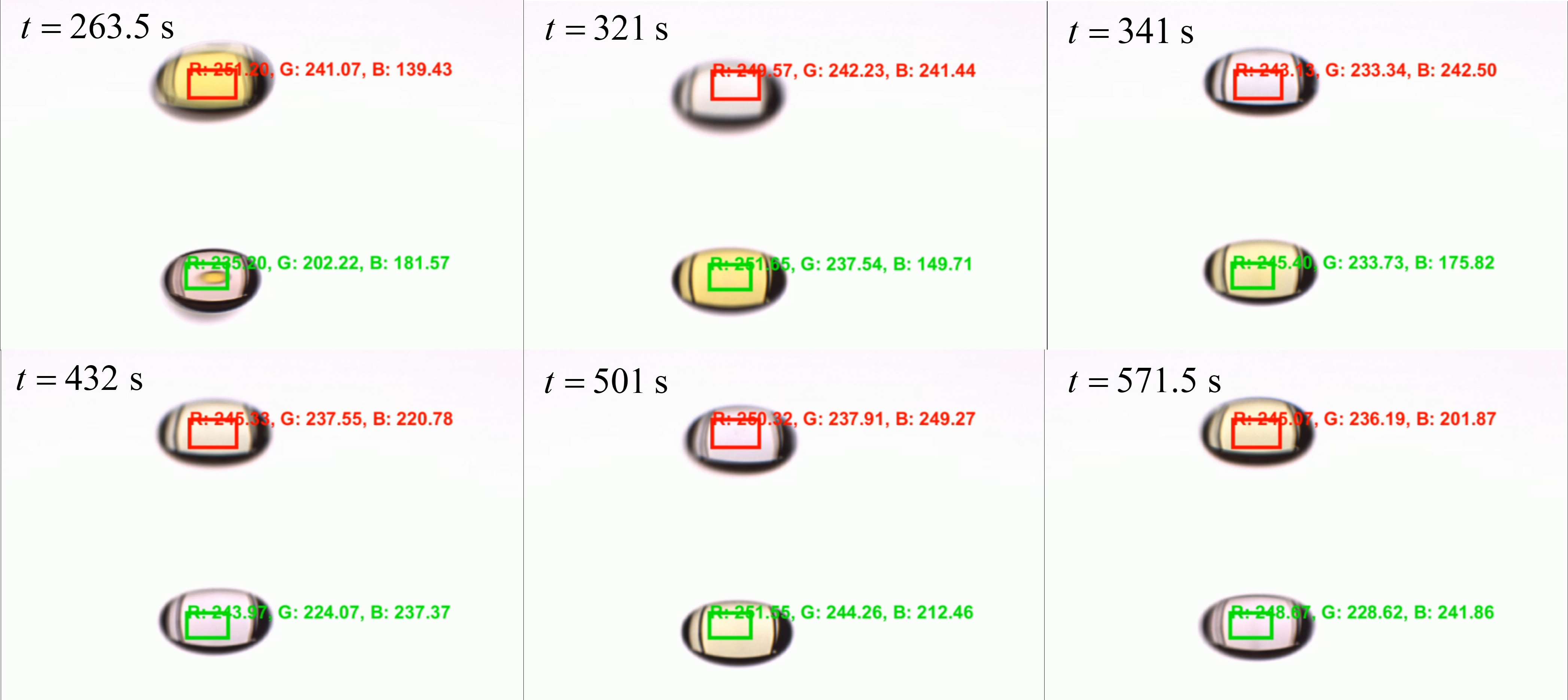


Fig. S13: Selected frames of oscillating reaction process. (See video S8 for the complete process). t = 263.5 s and t = 321 s correspond to the coalescence time of upper and lower droplets, respectively. The merged upper and lower droplets are denoted as droplet 1 and droplet 2 respectively. And the two fixed rectangles represent the region to calculate the average grayscale value for all the three colors (R: red; G: green; B: blue) with values indicating the current value of RGB.

We have scanned the acoustic filed shown in Fig. S14, the lower droplet is near a high-pressure region at *z* = -9 mm which may transfer more heat to the lower droplet so droplet 2 would have a higher temperature than droplet 1 and consume more chemicals before the final coalescence which may explain why the oscillating times in droplet 2 is always less than that in droplet 1.


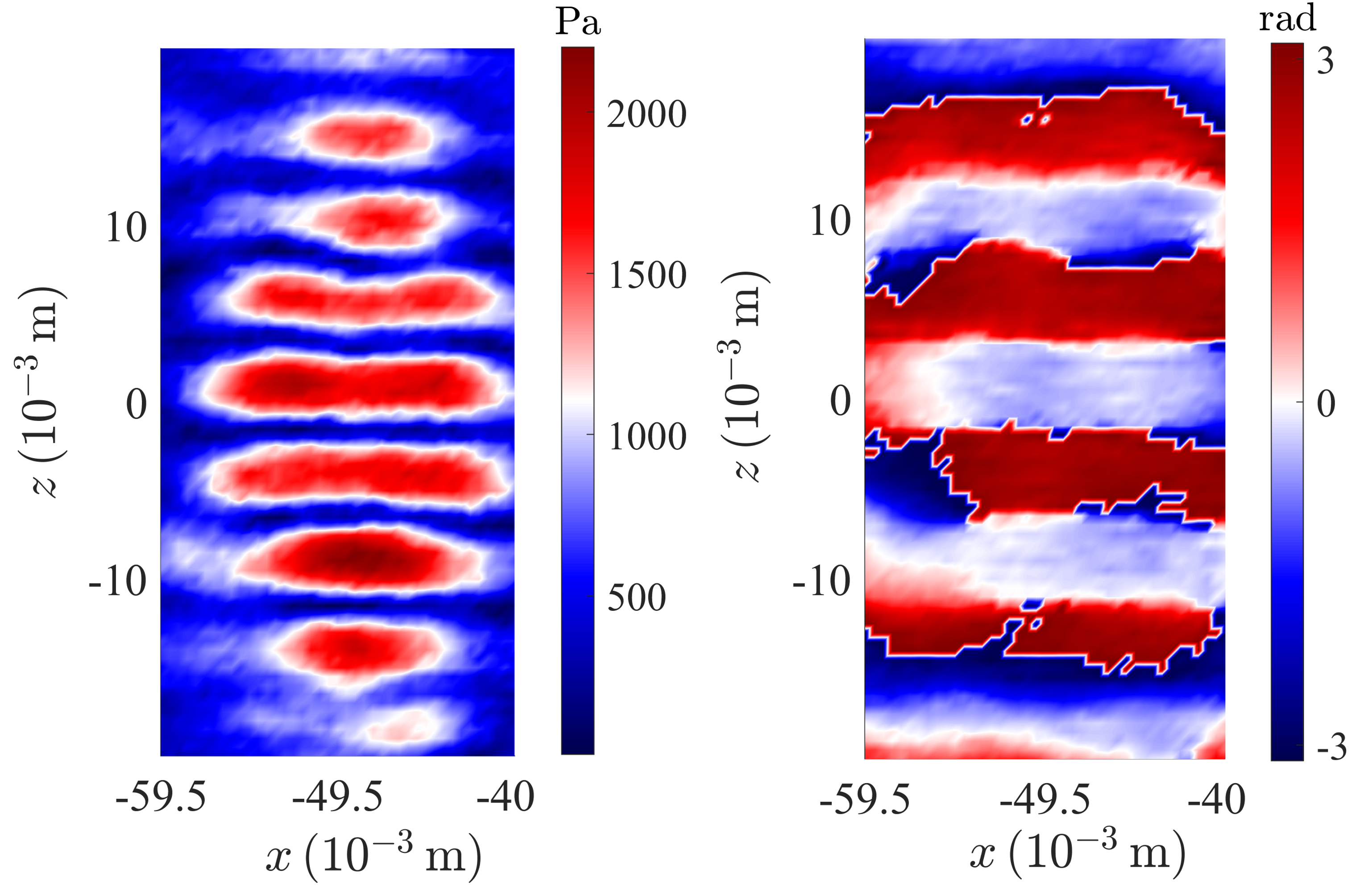


**Fig. S14: Scanned field of the final coalescence step where the left and right contour plots show pressure amplitude and phase, respectively.**

1. School of Chemistry, University of Birmingham, Birmingham, B15 2TT, UK. E-mail: [r.gupta.3@bham.ac.uk](mailto:r.gupta.3@bham.ac.uk) [↑](#footnote-ref-1)
2. No affiliation [↑](#footnote-ref-2)
